# Ferumoxytol-enhanced magnetic resonance angiography for the assessment of potential kidney transplant recipients

**DOI:** 10.1007/s00330-017-4934-5

**Published:** 2017-07-04

**Authors:** Sokratis Stoumpos, Martin Hennessy, Alex T. Vesey, Aleksandra Radjenovic, Ram Kasthuri, David B. Kingsmore, Patrick B. Mark, Giles Roditi

**Affiliations:** 1 Renal and Transplant Unit, Queen Elizabeth University Hospital, Glasgow, UK; 2 Institute of Cardiovascular and Medical Sciences, BHF Glasgow Cardiovascular Research Centre, University of Glasgow, Glasgow, G12 8TA UK; 3 Department of Radiology, Queen Elizabeth University Hospital, Glasgow, UK

**Keywords:** Magnetic resonance imaging, Kidney failure, chronic, Kidney transplantation, Angiography, Ferric compounds

## Abstract

**Objectives:**

Traditional contrast-enhanced methods for scanning blood vessels using magnetic resonance imaging (MRI) or CT carry potential risks for patients with advanced kidney disease. Ferumoxytol is a superparamagnetic iron oxide nanoparticle preparation that has potential as an MRI contrast agent in assessing the vasculature.

**Methods:**

Twenty patients with advanced kidney disease requiring aorto-iliac vascular imaging as part of pre-operative kidney transplant candidacy assessment underwent ferumoxytol-enhanced magnetic resonance angiography (FeMRA) between December 2015 and August 2016. All scans were performed for clinical indications where standard imaging techniques were deemed potentially harmful or inconclusive. Image quality was evaluated for both arterial and venous compartments.

**Results:**

First-pass and steady-state FeMRA using incremental doses of up to 4 mg/kg body weight of ferumoxytol as intravenous contrast agent for vascular enhancement was performed. Good arterial and venous enhancements were achieved, and FeMRA was not limited by calcification in assessing the arterial lumen. The scans were diagnostic and all patients completed their studies without adverse events.

**Conclusions:**

Our preliminary experience supports the feasibility and utility of FeMRA for vascular imaging in patients with advanced kidney disease due for transplant listing, which has the advantages of obtaining both arteriography and venography using a single test without nephrotoxicity.

***Key Points*:**

• *Evaluation of vascular disease is important in planning kidney transplantation.*

• *Standard vascular imaging methods are often problematic in kidney disease patients.*

• *FeMRA has the advantage of arteriography and venography in a single test.*

• *FeMRA is safe and non-nephrotoxic.*

• *FeMRA is not limited by arterial calcification.*

## Introduction

Kidney transplant candidates often require accurate assessment of their vascular anatomy before being wait-listed for transplantation. Imaging studies with vascular mapping can assess arterial and venous anatomy, thereby establishing whether kidney transplantation is possible, whether presurgical procedures are necessary, and which is the best surgical technique for each candidate. Conventional vascular imaging techniques are often problematic in kidney disease patients due to associated risks, invasiveness and imprecision. Computed tomography angiography (CTA) entails radiation exposure and the use of iodine-based contrast that may precipitate a deterioration in kidney function or even the need for renal replacement therapy [1, 2]. Magnetic resonance angiography (MRA) in patients with advanced kidney disease using linear gadolinium-based contrast agents (GBCAs) has been associated with the rare disease nephrogenic systemic fibrosis (NSF) [3]. Alternative imaging methods also have drawbacks; for example, Doppler ultrasound (US) is a potentially useful modality but can be difficult to interpret and is vulnerable to high inter- and intra-operator variability and patient habitus. This patient group has a higher risk of complications from conventional catheter-based x-ray angiography, and non-contrast-enhanced MRA (e.g. time-of-flight or phase-contrast techniques) is very time consuming for assessment of multidirectional flow with poor signal from deep vascular structures and at the sites of flow disturbance.

Ferumoxytol is a solution of ultrasmall superparamagnetic iron oxide (USPIO) particles encapsulated by a semisynthetic carbohydrate that prevents redistribution outside the vascular space. Ferumoxytol was initially developed as a magnetic resonance imaging (MRI) contrast agent in 2000 [4]. However, it was first licensed as a therapeutic agent for the treatment of iron deficiency anaemia (IDA) in patients with chronic kidney disease (CKD) [5]. Recently, ferumoxytol has regained appeal as an MRI contrast agent in patients with estimated glomerular filtration rates (eGFR) <30 ml/min/1.73 m^2^.

We report our findings in 20 patients with severely impaired renal function or on dialysis who underwent ‘off-label’ ferumoxytol-enhanced MRA (FeMRA) for vascular evaluation prior to being wait-listed for kidney transplantation.

## Materials and methods

### Study population

Patients more than 18 years of age with advanced kidney disease or on dialysis attending transplant assessment clinics in Glasgow and requiring vascular imaging prior to wait-listing for kidney transplantation were included in this series. Criteria for vascular imaging included intermittent claudication, known peripheral vascular disease (PVD) with previous angioplasty or revascularisation procedure or leg amputation, extensive disease in other vascular beds, and the presence of risk factors for PVD (diabetes, smoking, hypertension, obesity). In some instances, patients had FeMRA in addition to prior conventional CTA if the clinician felt that additional investigation was required to characterise the vessels before placement of a kidney graft. These were usually patients who had blooming artefacts from dense calcifications on initial CTA. Dialysis patients were included if there was evidence of extensive vascular calcification or they had residual renal function with a risk of accelerated decline in function after CTA. Patients with standard contraindications to MRI (such as non-MRI-compatible pacemakers, severe claustrophobia and metal in the eyes), history of allergic reaction to any intravenous iron product, any conditions associated with iron overload, and patients with active immune or inflammatory conditions (e.g. systemic lupus, rheumatoid arthritis) were excluded. This evaluation of the use of ferumoxytol as a new potential clinical service was approved by the Clinical Governance Committee of the Diagnostics Directorate of NHS Greater Glasgow & Clyde. In this context, the West of Scotland Research Ethics Committee ethics officer was consulted and confirmed that no formal ethics committee approval was required. Nevertheless, as this was an off-label use of the agent, informed consent was obtained from all subjects. Investigations were performed between 1 December 2015 and 1 August 2016.

### Baseline data

Age, gender, aetiology of established renal failure (ERF) and co-morbid conditions (including estimation of the Charlson Comorbidity Index (CCI) scores) were recorded. We also retrieved data on the last serum creatinine prior to FeMRA and calculated the eGFR using the six-variable Modification of Diet in Renal Disease (MDRD) formula [6].

CTA examinations were reviewed for patients who had had both imaging modalities performed as part of their assessment, and served as the reference standard for comparisons with FeMRA. Direct comparisons of predefined cross-sections of various vascular beds were performed to visually assess image quality. The CT and MR images were interpreted separately and the observer was blinded to patient identity, clinical findings and findings of the other imaging study.

### MRI protocol

Patients underwent MRA with ferumoxytol administered in an appropriate setting under supervision of trained medical personnel and were observed for a minimum period of 30 min following termination of ferumoxytol infusion.

All studies were performed on a 3.0 T Prisma MRI scanner (Magnetom, Siemens Medical Solutions, Erlangan, Germany) with local phased-array imaging coils using a standardised protocol similar to that of standard MRA studies with GBCAs. All patients were imaged in the supine position.

### Ferumoxytol administration

Ferumoxytol was infused intravenously through a 22-gauge intravenous catheter placed in the antecubital fossa of either arm. A total dose of 4 mg/kg of ferumoxytol (Feraheme; AMAG Pharmaceuticals, Inc., Cambridge, MA, USA) was delivered up to a maximum of 300 mg. The dosage of Feraheme is expressed in terms of mg of elemental iron, with each ml of Feraheme containing 30 mg of elemental iron. In all cases ferumoxytol was diluted to a concentration no greater than one part ferumoxytol to four parts 0.9% sodium chloride and was administered in four equally divided controlled infusions until the full standard dose was delivered. Ferumoxytol infusions were delivered by an MRI-compatible infusion pump for precise control over infusion rates. These were set at 1 ml/s of diluted ferumoxytol (equal to 6 mg/s of elemental iron) followed by 20 ml of 0.9% sodium chloride at a rate of 2 ml/s.

Patients were instructed to immediately alert the operator should they have any feelings of discomfort at any time. Patients were continuously monitored by pulse oximeter (measuring both heart rate and oxygen saturation) while in the MRI scanner and had blood pressure measured before and after infusions. Care was taken to ensure that the total cumulative dose was not administered in less than 20 min. Average scan duration was approximately 45 min.

### Image acquisition

Pre-contrast imaging was performed for morphological assessment. First-pass, breath-hold imaging with a dynamic contrast-enhanced technique was performed 15–20 s after delivery of the first infusion. These 3D, T1-weighted ultrafast spoiled gradient echo sequences were also obtained after each subsequent infusion and were considered first-pass (arterial phase) images. Further T1-weighted spoiled gradient echo sequences were used to obtain steady-state breath-hold (1 mm isotropic) and non-breath-hold higher spatial resolution (0.5 mm isotropic) abdominopelvic images after the first and each subsequent infusion until the full dose had been reached – each imaging initiated 3 min after contrast administration.

### Data analysis

Both arterial and venous structures were assessed. The examined arteries included abdominal aorta, coeliac artery, superior and inferior mesenteric arteries, renal arteries, and common, external and internal iliac arteries. The examined veins included inferior vena cava, renal veins, common, external and internal iliac veins, and common, superficial and deep femoral veins in the upper thigh. Visual assessment of the subjective image quality was performed independently by two radiologists (M.H and G.R.) with 5 years and >20 years of experience, respectively, in cardiovascular MR imaging. Image quality was assessed following administration of the full cumulative dose and was rated on a scale of 1–4 (1: non-diagnostic; 2: poor definition such that only gross features such as overall patency are evaluable; 3: good definition such that pathology can be confidently visualised or excluded; 4: excellent definition such that detailed anatomy is clearly visualised with sharp borders for all vessels) with respect to the abdominal aorta, the inferior vena cava and the common, external and internal iliac arteries and veins. The same vessels were used for comparisons with CTA studies. Interobserver agreement was calculated using weighted kappa statistics (<0.2: poor agreement; 0.2–0.4: fair agreement; 0.41–0.6: moderate agreement; 0.61–0.8: good agreement; 0.81–1.0: very good agreement). The IBM SPSS Statistics Package (version 22.0; SPSS, Inc., Armonk, NY, USA) was used for all analyses.

## Results

A total of 20 patients had FeMRA as part of their pre-operative kidney transplant assessment. Eight (40%) were haemodialysis patients. The mean age was 61.2 (standard deviation (SD) 11.5) years, 85% were men and 60% were diabetic. All patients had a high burden of co-morbidity and only one patient did not have diabetes, documented atherosclerotic vascular disease or heart failure. Nineteen (95%) patients had a high CCI (greater than 2) and, of those, 50% had a very high CCI (greater than 4) (Table 1). In four patients, arterial vascular anatomy had been examined with CTA prior to FeMRA.

**Table 1:** Demographic and clinical characteristics of patients

Age (y), mean (SD)	61.2 (11.5)
Male sex, n (%)	17 (85.0)
Cause of ERF	
Diabetes, n (%) Renovascular, n (%) Other^a^, n (%) Unknown aetiology, n (%)	9 (45.0) 4 (20.0) 4 (20.0) 3 (15.0)
Co-morbidity	
Diabetes, n (%) Vascular disease^b^, n (%) Heart failure, n (%) None of the above, n (%)	12 (60.0) 16 (80.0) 3 (15.0) 1 (5.0)
Charlson Comorbidity Index (CCI) score	
CCI 1–2, n (%) CCI 3–4, n (%) CCI ≥5, n (%)	1 (5.0) 9 (45.0) 10 (50.0)
eGFR at time of scan^c^ (ml/min/1.73 m^2),^ mean (SD) Dialysis, n (%) <15, n (%) 15–29, n (%)	14.0 (4.5) 8 (40.0) 8 (40.0) 4 (20.0)

^a^ Glomerulonephritis (n = 2), autosomal dominant polycystic kidney disease (n = 1), congenital renal dysplasia (n = 1)

^b^ Coronary artery disease, cerebrovascular disease, peripheral vascular disease

^c^ Excludes eight patients on dialysis

*SD* standard deviation, *ERF* established renal failure, *eGFR* estimated glomerular filtration rate; *CT* computed tomography

All subjects completed first-pass and steady-state MRA acquisitions with ferumoxytol enhancement. There were no adverse events associated with ferumoxytol administration. The imaging parameters for the post-contrast breath-hold MRA acquisitions are listed in Table 2.

**Table 2:** Pulse sequence parameters for the T1-weighted 3D spoiled gradient echo sequences (post-contrast)

Repetition time (ms)	2.88
Echo time (ms)	1.04
Flip angle (°)	20
Slice thickness (mm)	1.0
Voxel dimensions (mm)	1.0 x 1.0 x 1.0
Field of view (mm)	400
Acquisition matrix	243 x 384
Timing of sequence^a^ (s)	60
Acquisition Time (s)	18
Signal averages	1
Mean volume thickness	112
Bandwidth (Hz/pixel)	300
Parallel imaging acceleration factor	3

^a^ After start of contrast infusion

Image quality on steady-state acquisitions was scored as grade 4 in 245 of 320 (76.6%; 90% confidence interval (CI) 72–79) and grade 3 in 75 of 320 (23.4%) vascular sections (at least diagnostic quality) when assessing the arterial and venous vasculature by both readers. There were no arterial anatomical characteristics or lesions of clinical significance that were identified on CTA and not on FeMRA, and vice versa. There was very good agreement on all individual assessments of image quality (kappa = 0.85 on assessment of the arteries; kappa = 0.76 on assessment of the veins; and kappa = 0.93 on assessment of the arteries on CTA vs. FeMRA).

**Fig. 1:**
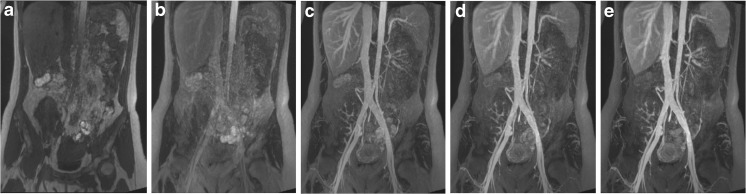
Arterial phase maximum intensity projection (MIP) images of abdominal and aorto-iliac vasculature after each increment of ferumoxytol. (**A**) Pre-contrast and after administration of (**B**) 1 mg/kg, (**C**) 2 mg/kg, (**D**) 3 mg/kg and (**E**) 4 mg/kg of ferumoxytol. Both arterial and venous compartments enhance due to contrast pooled intravascularly from previous infusions

**Fig. 2:**
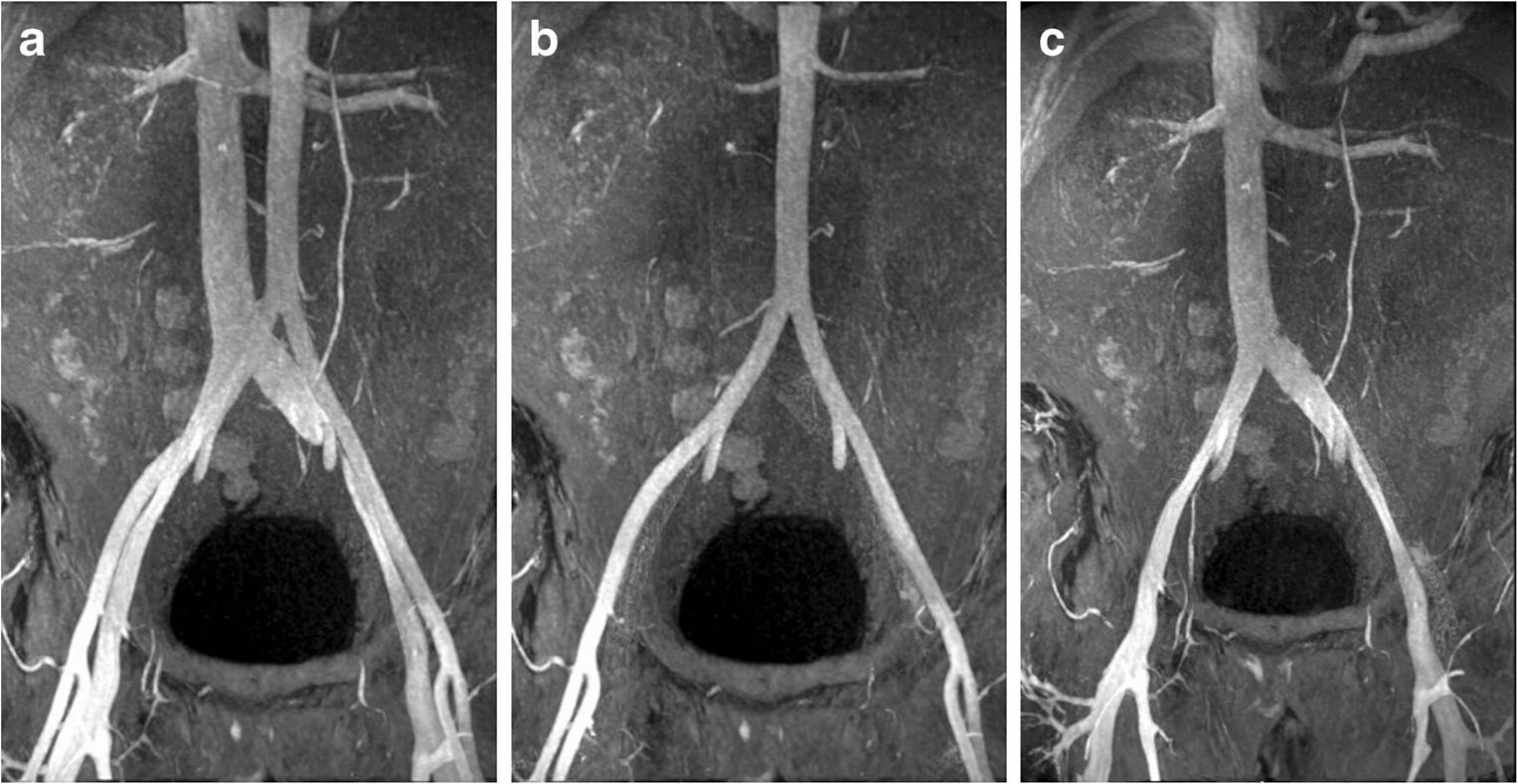
Ferumoxytol-enhanced magnetic resonance angiography (FeMRA) of abdominal and aorto-iliac vasculature in patient referred for kidney transplant evaluation. (**A**) Steady-state acquisition showing enhancement of both arterial and venous vasculature. (**B**) First-pass imaging showing selective arterial enhancement (arteriography). (**C**) Steady-state acquisition showing selective venous enhancement after subtraction of the arterial compartment (venography)

Following each dose increment, signal intensity and image quality were improved in both arterial and venous compartments (Fig. 1). First-pass acquisitions showed selective arterial enhancement, with both arterial and venous enhancement on delayed acquisitions (Fig. 2A, B). Selective venous imaging was obtained by subtraction of arterial phase images from steady-state images (Fig. 2C).

**Fig. 3:**
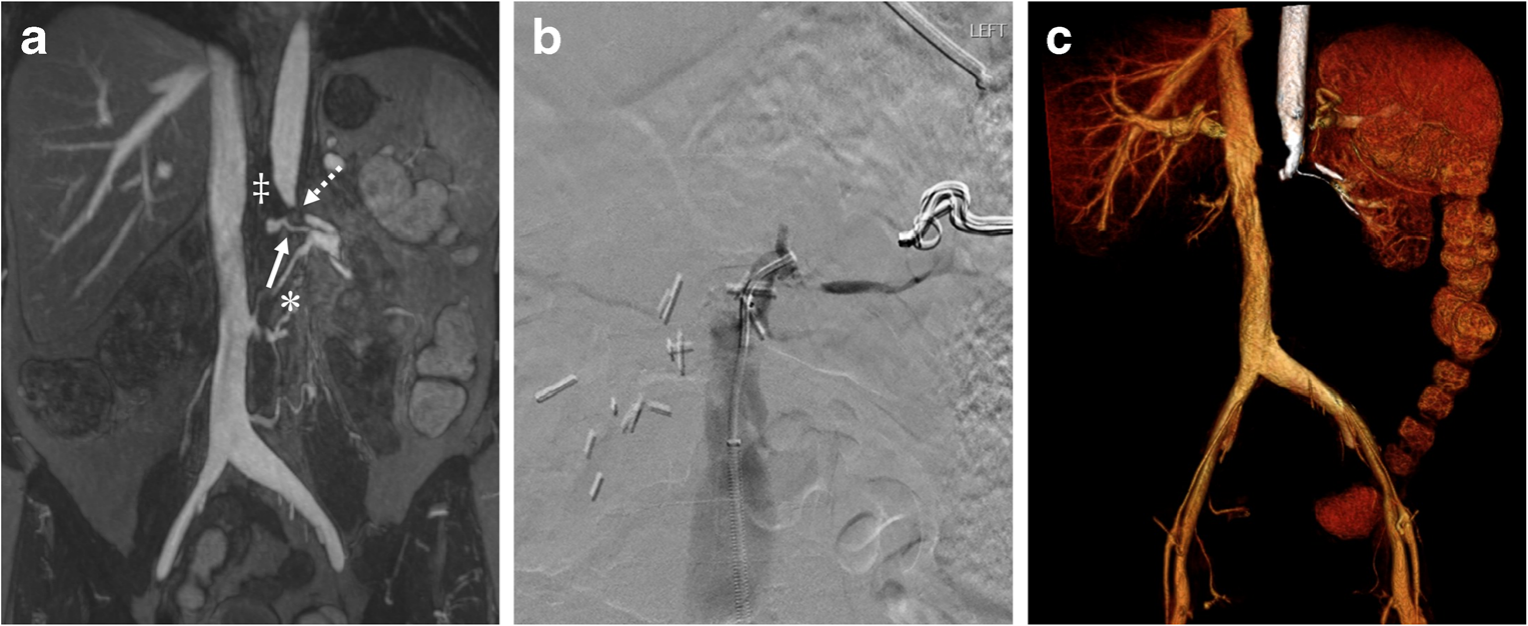
(**A**) Ferumoxytol-enhanced magnetic resonance angiography (FeMRA) in an individual with a tightly stenosed left main renal artery (dashed arrow), a small patent accessory renal artery (solid arrow), and an occluded right renal artery (‡) and infrarenal abdominal aorta (*). (**B**) Digital subtraction angiography (DSA) after aorto-bifemoral stent grafting showing the tightly stenosed left main renal artery. (**C**) Three-dimensional reconstruction of aorta and inferior vena cava at the level of bifurcation of renal arteries

**Fig. 4:**
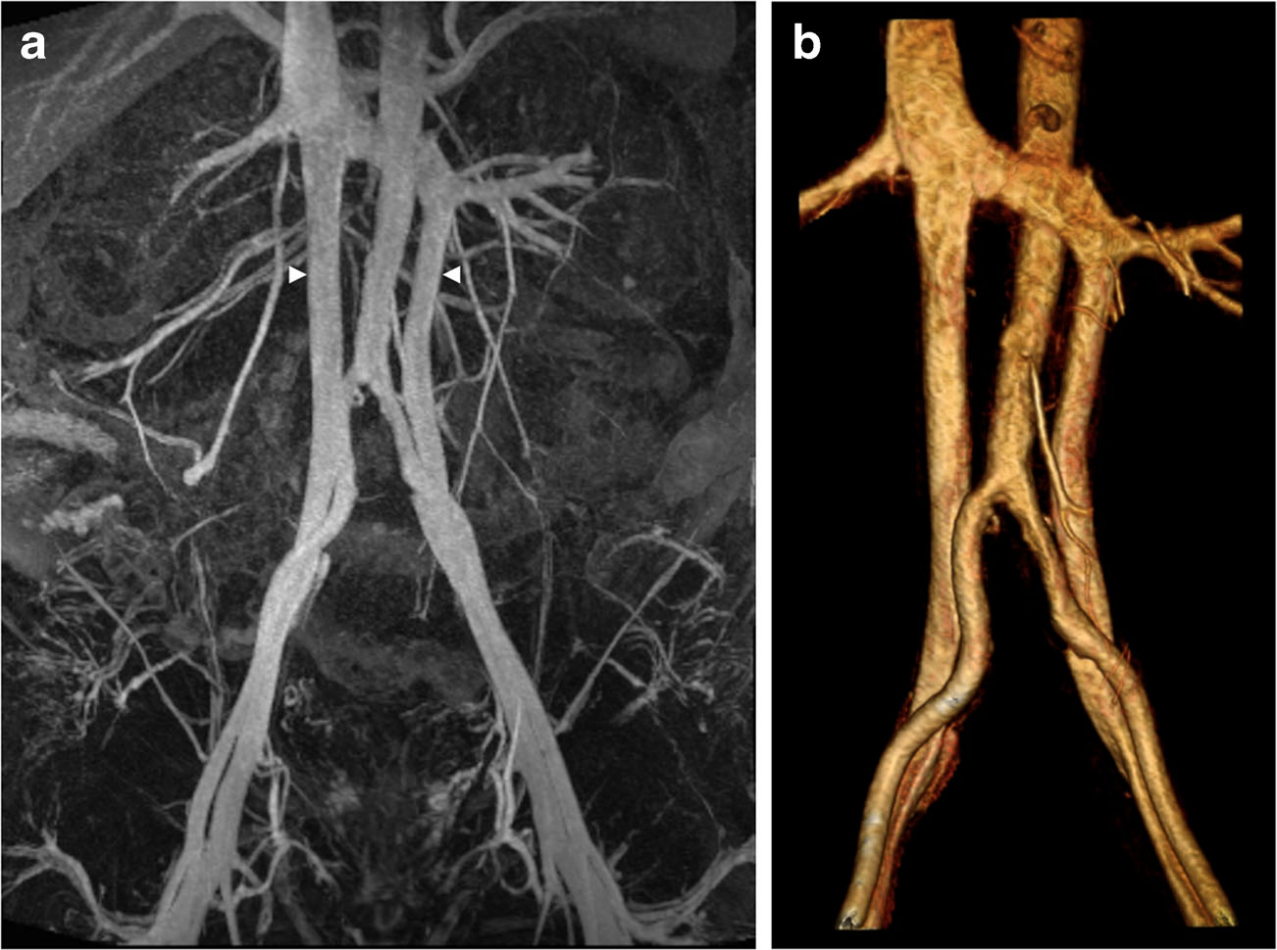
(**A**) Steady-state acquisitions with ferumoxytol-enhanced magnetic resonance angiography (FeMRA) in an individual with an anatomical variant of a dual inferior vena cava (arrowheads). (**B**) Three-dimensional reconstruction of abdominal vasculature at the level of the inferior vena cava duplication

**Fig. 5:**
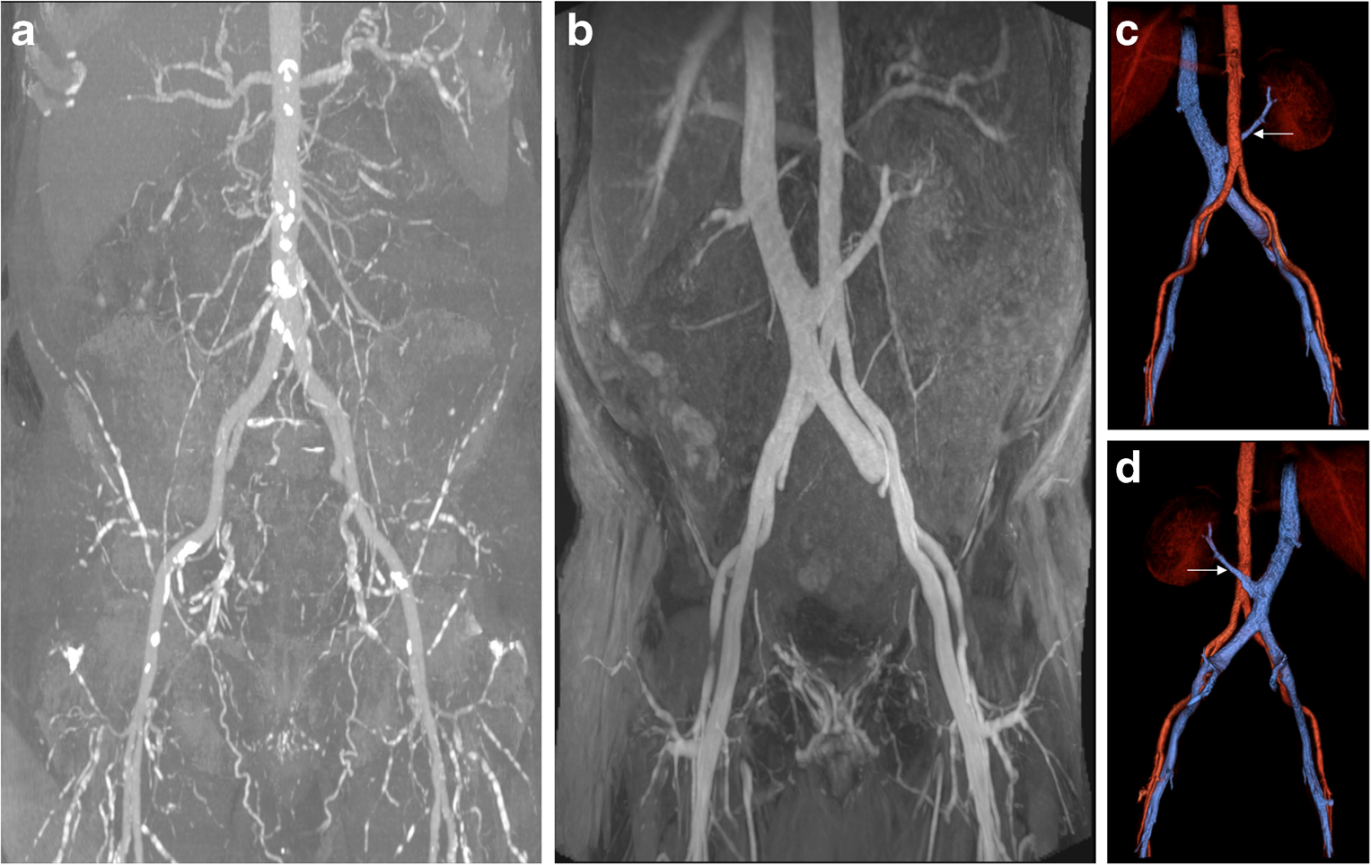
Abdominal and aorto-iliac vasculature in a patient referred for kidney transplant assessment. (**A**) Coronal CT angiogram through the upper abdomen showing foci of calcification at the aortic sector and iliac arteries. (**B**) Steady-state FeMRA of the same patient with synchronous illustration of aorto-iliac sector and inferior vena cava. (**C**) Anterior and (**D**) posterior views of three-dimensional reconstruction of abdominal and aorto-iliac vasculature showing an anatomical variant of a low-lying retro-aortic left renal vein (arrow)

**Fig. 6:**
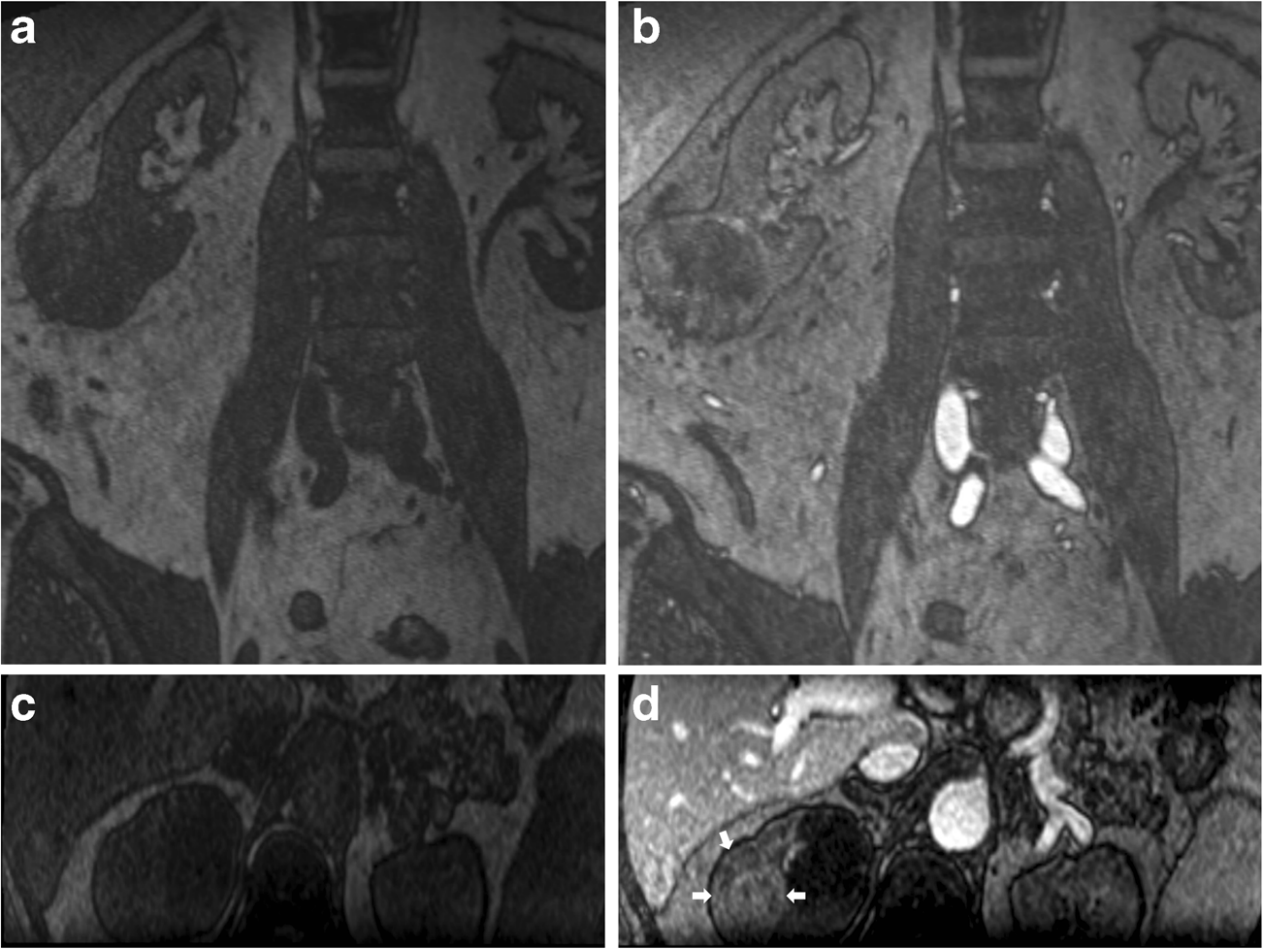
Ferumoxytol-enhanced magnetic resonance angiography (FeMRA) showing incidental renal tumours in two patients. (**A**) Coronal pre- and (**B**) post-contrast T1-weighted first-pass acquisitions through the upper abdomen showing tumour with peripheral enhancement and central necrosis originating from the lower pole of the right kidney. (**C**) Transverse pre- and (**D**) post-contrast T1-weighted first-pass acquisitions through the upper abdomen from another patient showing a large partially enhancing right renal mass (arrows)

A patient with extensive vascular disease was found to have bilateral renal artery and infrarenal abdominal aorta stenosis/occlusions and underwent aorto-bifemoral stent grafting followed by stenting of the left main renal artery (Fig. 3). Interesting anatomical variants were illustrated in a patient with a dual inferior vena cava (Fig. 4) and one with a retro-aortic left renal vein (Fig. 5). Two patients in this series were found to have incidental complex renal cysts of the native kidneys that had enhancing components with ferumoxytol (Fig. 6); both of these were later confirmed to be renal cell carcinomas on histology.

## Discussion

We applied an MRA technique using intravenous iron as ferumoxytol to assess vessel characteristics, patency and course in a series of patients with severely impaired renal function or on dialysis whilst planning renal transplantation. Study participants underwent FeMRA as part of their clinical care instead of or in addition to standard imaging tests. We administered a total of 4 mg/kg of diluted ferumoxytol in divided infusions and achieved good signal intensity and image quality with absence of any adverse events. The information gained from synchronous depiction of arterial and venous compartments using a single investigation is essential when planning kidney transplantation. In a subgroup of patients who had both FeMRA and CTA performed, FeMRA was not limited by calcification in assessing the arterial lumen and it was better for venous evaluation compared to CTA.

Vascular disease is almost universal in potential kidney transplant recipients, the evaluation of which is important in planning transplantation. Doppler US is often not appropriate for evaluation of the deep vessels of the abdomen and pelvis and calcification further interferes with US assessment of the arterial vasculature. CTA of the aorto-iliac vasculature has been shown to allow better recipient selection and accurate planning for the arterial anastomosis [7], but is limited by calcification and also does not robustly evaluate venous anatomy. This latter limitation is of particular importance as frequently indwelling femoral vein catheters will have been employed and may have resulted in venous stenosis. MRI is limited by the concerns around potential development of NSF when high-risk linear chelate GBCAs are used. Practitioners are advised against the use of all linear GBCAs in patients with acute kidney injury or CKD with an eGFR <30 ml/min/1.73 m^2^. On the other hand, although NSF seems not to be a risk with more stable gadolinium agents such as the cyclic chelates, residual concerns have constrained use. Lastly, conventional invasive angiography is usually reserved for use in the setting of possible endovascular intervention or targeted angiography when lower volumes of iodine-based contrast medium can be used in patients with CKD.

The risks of post-contrast acute kidney injury (AKI) have been questioned in recent publications [8, 9], although this remains controversial. Notably, in a series of similar design retrospective studies, McDonald and colleagues examined the rate of AKI in the 24–72 h after intravenous contrast-enhanced CT or non-contrast-enhanced CT imaging [10–13]. Although diminished eGFR was associated with an increased risk of AKI and patients who developed AKI had higher rates of dialysis and mortality, the occurrence of these outcomes did not differ significantly between the contrast and non-contrast group in these studies. Nevertheless, these studies should be interpreted with caution as despite matching, in a retrospective study there may have been bias present in selection of the controls, which may erroneously underestimate or even obscure the real effects of contrast medium. Because of its large molecular weight of approximately 750 kD, ferumoxytol is not filtered by the glomerulus, but rather is removed from the circulation via phagocytosis by macrophages, and subsequently broken down. Following macrophage breakdown, the remaining iron oxide particles are taken up by the reticuloendothelial system, in particular the liver, spleen and bone marrow. The distribution of USPIO and conventional GBCAs is different, with the former remaining in the blood pool prior to largely redistributing to the reticuloendothelial system, whereas the latter distributes into the extracellular space after a relatively short time within the blood pool. With ferumoxytol, arteries and veins can be selectively depicted in a single examination. Additionally, the prolonged intravascular half-life (>14 h) of ferumoxytol allows for a longer time window for data acquisition, higher spatial resolution during the equilibrium phase and repeat imaging, if necessary, with negligible loss of intravascular signal intensity [14–16]. On the downside, the agent can be present in the blood pool for weeks following administration and months in the reticuloendothelial system, potentially complicating the appearance of follow-up studies [17]. A previously described susceptibility artefact mimicking vascular thrombosis [18] related to higher concentrations of ferumoxytol was not observed in our study. In that study the authors used imaging sequences primarily intended for parenchymal evaluation rather than MR angiography, higher concentrations of ferumoxytol (up to 0.43 vs. 0.2 ml/ml total volume), longer echo times (1.8 vs. 1.0 ms) and slower infusion rates (2 vs. 1 ml/s), all potentially associated with susceptibility artefact.

Ferumoxytol has a good safety profile and no known long-term toxicity [19]. Adverse events with regard to cardiovascular, infectious and mortality outcomes have been shown to be similar compared with the more commonly used intravenous iron formulations (iron sucrose and ferric gluconate) [20]. Although the reported incidence of adverse reactions is higher with ferumoxytol than with GBCAs, the rates of these in clinical trials and post-marketing safety data on therapeutic use of ferumoxytol are very low [5, 21–23]. Most reported adverse events were mild, transient and typically associated with the infusion process, although mild arthralgia/myalgia and headaches occurred up to 48 h post-infusion in one study [23], where 1.02 g of ferumoxytol was administered over 15 min. Serious adverse events included hypersensitivity [22, 24] and hypotension [24] with a pooled aggregate rate of anaphylaxis of 0.03% (3/10,425) based on published studies [5, 21, 22, 24]. The main concern has been reported cases of anaphylactic-type reactions with therapeutic bolus injections of undiluted compound leading to the recent recommendations for controlled infusion of dilute ferumoxytol. Rather than true allergy it is thought this is a chemotoxic phenomenon related to high concentrations of the iron compound when infused rapidly interacting with mast cells in the vascular wall [25]. Since 2009, approximately 1.2 million therapeutic doses of ferumoxytol have been administered. In March 2015, the US Food and Drug Administration (FDA) Adverse Event Reporting System reported 79 anaphylactic reactions, of which 18 were fatal. These deaths resulted in a boxed warning in March 2015 (http://www.fda.gov/Drugs/DrugSafety/ucm440138.htm). Twenty-four percent of these patients had multiple drug allergies, and nearly half of these anaphylactic reactions occurred within 5 min of administration. This rate of adverse events is lower than the rates initially reported in Phase II–III clinical trials. When given for treatment of IDA, the licensed dose of ferumoxytol is an initial 510-mg dose administered as an intravenous infusion, followed by a second 510-mg dose 3–8 days later. We used only a fraction of the therapeutic dose (approximately a quarter of the full dose for a 70-kg adult), much-diluted and slowly infused (in 5-min intervals between pulsed infusions) to minimise the risk of reaction, and this was proven more than adequate for vascular imaging. It is important to note that the above risks have parity with the other commonly administered intravenous radiological contrast media (whether iodine or gadolinium-based), therefore patients were not exposed to disproportionate risk. Hence, ferumoxytol has the potential to be used for clinical vascular imaging beyond investigational use in patients with contraindications to GBCAs, such as renal failure and known GBCAs allergies.

Dose-related efficacy studies are lacking, hence different doses have been reported in the literature, ranging from 120 mg of elemental iron in a bolus solution for angiographic assessment of arteriovenous fistulae [26] to 6 mg/kg for kidney mapping [27]. We used a comprehensive protocol administering a total of 4 mg/kg of ferumoxytol diluted fourfold with normal saline and delivered in four equally divided infusions over a minimum of 20 min and we observed no significant blunting in signal intensity.

Others have studied MRA using ferumoxytol as contrast in patients with kidney disease. Sigovan et al. [26] found that FeMRA provided better image quality and reduced flow artefacts compared to non-contrast MRA in dialysis fistula evaluation, with a much shorter acquisition time (19 vs. 270 s). FeMRA has been used in kidney transplant recipients with graft dysfunction, where steady-state imaging was better for evaluation of transplant vasculature compared to first-pass imaging [28] and better for detection of graft artery abnormalities compared to US [29]. When compared with digital subtraction angiography (DSA), FeMRA was similarly sensitive and accurate in assessing the severity of transplant renal artery stenosis [30]. In a comparative study of ferumoxytol-enhanced versus gadofosveset-enhanced MR venography for abdominopelvic and lower extremity venous assessment, there was no difference in signal intensity between the two techniques [31]. In two paediatric cohorts of 30 children with CKD who needed detailed vascular mapping for various indications (i.e. vascular access planning or complication, pre- or post-kidney transplant evaluation), FeMRA examinations were reported to be diagnostic and safe [32, 33]. Apart from vascular imaging, ferumoxytol-based imaging has been successfully used for the delineation of primary pancreatic tumours [34] and as an ionising radiation-free staging method in children and young adults with malignant lymphomas and sarcomas [35]. In our study, ferumoxytol-enhancing complex renal cysts were detected in two patients; both proved to be renal cell carcinomas.

The main limitations of this study are the single-centre design with a relatively small number of patients and lack of consistent reference standard. However, the purpose of this study was to address the technical feasibility of acquiring adequate abdominal and aorto-iliac vascular enhancement with a non-nephrotoxic, high relaxivity contrast agent in patients before wait-listing for kidney transplantation. Four patients had both CTA and FeMRA performed and although some comparisons were made, the study was not designed to estimate accuracy, sensitivity and specificity of the two different imaging techniques. A formal prospective study directly comparing the diagnostic accuracy of FeMRA versus CTA is currently recruiting in our centre (Clinicaltrials.gov identifier: NCT02997046).

High concentrations of the agent can cause artefacts because of signal loss through T2* shortening, and care must be taken to inject a low enough concentration to avoid this pitfall. Therefore, we used small cumulative increments of ferumoxytol but in this preliminary report on 20 subjects the number of patients was not large enough to establish the definitive optimal (minimum) dose required to achieve adequate diagnostic accuracy. However, our preliminary findings indicate that the administration of doses >3 mg/kg does not improve image quality (unpublished data). Despite being a case series, sequential enrolment minimised selection bias. In addition, differences in exposure to ferumoxytol were minimised by the use of a consistent protocol with standardised infusion rates and imaging parameters. Using a consistent dosing regimen for contrast administration, we have shown that ferumoxytol-based vascular imaging has the potential to offer a clinically useful and reliable alternative in renal patients in whom standard imaging methods cannot be used.

## Abbreviations

CCI: Charlson Comorbidity Index
ERF: Established renal failure
FeMRA: Ferumoxytol-enhanced magnetic resonance angiography
MDRD: Modification of diet in renal disease
NSF: Nephrogenic systemic fibrosis
USPIO: Ultrasmall superparamagnetic iron oxide

## References

[1] Davenport MS, Khalatbari S, Dillman JR, Cohan RH, Caoili EM, Ellis JH (2013). Contrast material-induced nephrotoxicity and intravenous low-osmolality iodinated contrast material. Radiology.

[2] Parfrey PS, Griffiths SM, Barrett BJ (1989). Contrast material-induced renal failure in patients with diabetes mellitus, renal insufficiency, or both. A prospective controlled study. N Engl J Med.

[3] Collidge TA, Thomson PC, Mark PB (2007). Gadolinium-enhanced MR imaging and nephrogenic systemic fibrosis: retrospective study of a renal replacement therapy cohort. Radiology.

[4] Prince MR, Zhang HL, Chabra SG, Jacobs P, Wang Y (2003). A pilot investigation of new superparamagnetic iron oxide (ferumoxytol) as a contrast agent for cardiovascular MRI. J Xray Sci Technol.

[5] Macdougall IC, Strauss WE, McLaughlin J, Li Z, Dellanna F, Hertel J (2014). A randomized comparison of ferumoxytol and iron sucrose for treating iron deficiency anemia in patients with CKD. Clin J Am Soc Nephrol: CJASN.

[6] Levey AS, Bosch JP, Lewis JB, Greene T, Rogers N, Roth D (1999). A more accurate method to estimate glomerular filtration rate from serum creatinine: a new prediction equation. Modification of Diet in Renal Disease Study Group. Ann Intern Med.

[7] Andres A, Revilla Y, Ramos A (2003). Helical computed tomography angiography is the most efficient test to assess vascular calcifications in the iliac arterial sector in renal transplant candidates. Transplant Proc.

[8] Wilhelm-Leen E, Montez-Rath ME, Chertow G (2017). Estimating the risk of radiocontrast-associated nephropathy. J Am Soc Nephrol.

[9] McDonald JS, McDonald RJ, Comin J (2013). Frequency of acute kidney injury following intravenous contrast medium administration: a systematic review and meta-analysis. Radiology.

[10] McDonald JS, McDonald RJ, Carter RE, Katzberg RW, Kallmes DF, Williamson EE (2014). Risk of intravenous contrast material-mediated acute kidney injury: a propensity score-matched study stratified by baseline-estimated glomerular filtration rate. Radiology.

[11] McDonald RJ, McDonald JS, Carter RE (2014). Intravenous contrast material exposure is not an independent risk factor for dialysis or mortality. Radiology.

[12] McDonald RJ, McDonald JS, Bida JP (2013). Intravenous contrast material-induced nephropathy: causal or coincident phenomenon?. Radiology.

[13] McDonald JS, McDonald RJ, Lieske JC (2015). Risk of acute kidney injury, dialysis, and mortality in patients with chronic kidney disease after intravenous contrast material exposure. Mayo Clin Proc.

[14] Bremerich J, Bilecen D, Reimer P (2007). MR angiography with blood pool contrast agents. Eur Radiol.

[15] Ersoy H, Jacobs P, Kent CK, Prince MR (2004). Blood pool MR angiography of aortic stent-graft endoleak. AJR Am J Roentgenol.

[16] Bashir MR, Bhatti L, Marin D, Nelson RC (2015). Emerging applications for ferumoxytol as a contrast agent in MRI. J Magn Reson Imaging: JMRI.

[17] Storey P, Lim RP, Chandarana H (2012). MRI assessment of hepatic iron clearance rates after USPIO administration in healthy adults. Investig Radiol.

[18] Fananapazir G, Marin D, Suhocki PV, Kim CY, Bashir MR (2014). Vascular artifact mimicking thrombosis on MR imaging using ferumoxytol as a contrast agent in abdominal vascular assessment. J Vasc Interv Radiol: JVIR.

[19] Vasanawala SS, Nguyen KL, Hope MD (2016). Safety and technique of ferumoxytol administration for MRI. Magn Reson Med.

[20] Airy M, Mandayam S, Mitani AA (2015). Comparative outcomes of predominant facility-level use of ferumoxytol versus other intravenous iron formulations in incident hemodialysis patients. Nephrol Dial Transplant: Off Publ Eur Dial Transplant Assoc Eur Ren Assoc.

[21] Hetzel D, Strauss W, Bernard K, Li Z, Urboniene A, Allen LF (2014). A Phase III, randomized, open-label trial of ferumoxytol compared with iron sucrose for the treatment of iron deficiency anemia in patients with a history of unsatisfactory oral iron therapy. Am J Hematol.

[22] Vadhan-Raj S, Strauss W, Ford D (2014). Efficacy and safety of IV ferumoxytol for adults with iron deficiency anemia previously unresponsive to or unable to tolerate oral iron. Am J Hematol.

[23] Auerbach M, Strauss W, Auerbach S, Rineer S, Bahrain H (2013). Safety and efficacy of total dose infusion of 1,020 mg of ferumoxytol administered over 15 min. Am J Hematol.

[24] Schiller B, Bhat P, Sharma A (2014). Safety and effectiveness of ferumoxytol in hemodialysis patients at 3 dialysis chains in the United States over a 12-month period. Clin Ther.

[25] Bircher AJ, Auerbach M (2014). Hypersensitivity from intravenous iron products. Immunol Allergy Clin N Am.

[26] Sigovan M, Gasper W, Alley HF, Owens CD, Saloner D (2012). USPIO-enhanced MR angiography of arteriovenous fistulas in patients with renal failure. Radiology.

[27] Hedgire SS, McDermott S, Wojtkiewicz GR, Abtahi SM, Harisinghani M, Gaglia JL (2014). Evaluation of renal quantitative T2* changes on MRI following administration of ferumoxytol as a T2* contrast agent. Int J Nanomedicine.

[28] Corwin MT, Fananapazir G, Chaudhari AJ (2016). MR angiography of renal transplant vasculature with ferumoxytol: comparison of high-resolution steady-state and first-pass acquisitions. Acad Radiol.

[29] Bashir MR, Jaffe TA, Brennan TV, Patel UD, Ellis MJ (2013). Renal transplant imaging using magnetic resonance angiography with a nonnephrotoxic contrast agent. Transplantation.

[30] Fananapazir G, Bashir MR, Corwin MT, Lamba R, Vu CT, Troppmann C (2017). Comparison of ferumoxytol-enhanced MRA with conventional angiography for assessment of severity of transplant renal artery stenosis. J Magn Reson Imaging: JMRI.

[31] Bashir MR, Mody R, Neville A (2014). Retrospective assessment of the utility of an iron-based agent for contrast-enhanced magnetic resonance venography in patients with endstage renal diseases. J Magn Reson Imaging: JMRI.

[32] Nayak AB, Luhar A, Hanudel M (2015). High-resolution, whole-body vascular imaging with ferumoxytol as an alternative to gadolinium agents in a pediatric chronic kidney disease cohort. Pediatr Nephrol.

[33] Luhar A, Khan S, Finn JP (2016). Contrast-enhanced magnetic resonance venography in pediatric patients with chronic kidney disease: initial experience with ferumoxytol. Pediatr Radiol.

[34] Hedgire SS, Mino-Kenudson M, Elmi A, Thayer S, Fernandez-del Castillo C, Harisinghani MG (2014). Enhanced primary tumor delineation in pancreatic adenocarcinoma using ultrasmall super paramagnetic iron oxide nanoparticle-ferumoxytol: an initial experience with histopathologic correlation. Int J Nanomedicine.

[35] Klenk C, Gawande R, Uslu L (2014). Ionising radiation-free whole-body MRI versus (18)F-fluorodeoxyglucose PET/CT scans for children and young adults with cancer: a prospective, non-randomised, single-centre study. Lancet Oncol.

